# Cardiac CT blooming artifacts: clinical significance, root causes and potential solutions

**DOI:** 10.1186/s42492-022-00125-0

**Published:** 2022-12-09

**Authors:** Jed D. Pack, Mufeng Xu, Ge Wang, Lohendran Baskaran, James Min, Bruno De Man

**Affiliations:** 1 GE Research, Niskayuna, NY 12309 USA; 2 Rensselaer Polytechnic Institute, Troy, NY 12180 USA; 3 Weill Cornell Medicine, New York, NY 10065 USA; 4 National Heart Centre, Singapore, 169609 Singapore; 5 Cleerly, New York, NY 10065 USA

**Keywords:** Cardiac CT, Coronary artery imaging, Calcium blooming, In-stent restenosis, Partial volume, Motion artifacts, Beam hardening

## Abstract

This review paper aims to summarize cardiac CT blooming artifacts, how they present clinically and what their root causes and potential solutions are. A literature survey was performed covering any publications with a specific interest in calcium blooming and stent blooming in cardiac CT. The claims from literature are compared and interpreted, aiming at narrowing down the root causes and most promising solutions for blooming artifacts. More than 30 journal publications were identified with specific relevance to blooming artifacts. The main reported causes of blooming artifacts are the partial volume effect, motion artifacts and beam hardening. The proposed solutions are classified as high-resolution CT hardware, high-resolution CT reconstruction, subtraction techniques and post-processing techniques, with a special emphasis on deep learning (DL) techniques. The partial volume effect is the leading cause of blooming artifacts. The partial volume effect can be minimized by increasing the CT spatial resolution through higher-resolution CT hardware or advanced high-resolution CT reconstruction. In addition, DL techniques have shown great promise to correct for blooming artifacts. A combination of these techniques could avoid repeat scans for subtraction techniques.

## Introduction

Cardiac CT angiography (CCTA) has come a long way since its introduction near the turn of the century. Pioneering work in imaging the heart using 4-slice, 0.5 s-gantry CT scanners required long breath holds (30 s), gave spatial resolution of between 1.3 and 3.3 mm in the axial direction, and delivered a relatively high radiation dose due to the retrospective nature of the gating [1–3]. Multi-slice scanner technology developed quickly thereafter. By 2005, 64-slice scanners with faster gantry rates (approximately 0.35 s), higher coverage (approximately 4 cm), better spatial resolution (approximately 0.5 mm), and prospective gating techniques were available and enabled robust coronary imaging in a routine clinical setting in as few as five heartbeats [4]. When combined with the administration of heart rate lowering medications, 64-slice scanners proved to be quite effective at ruling out coronary artery disease (CAD) in many patients. Remaining challenges included motion artifacts, banding artifacts (due to misregistration and contrast dynamics), calcium blooming, and noise (for high body mass index patients).

In recent years, great strides have been made toward addressing the four challenges listed above. Temporal resolution has been improved as gantry periods have fallen further (to approximately one fourth s/rot), but progress along this direction has slowed since doubling the gantry speed quadruples the G-forces and places immense mechanical requirements on the gantry. Gantry speed improvements alone cannot meet the high demands of imaging the coronaries robustly at all cardiac phases. The temporal resolution has been further improved by roughly a factor of two by adding a second CT beamline (source/detector pair) on the gantry of some scanners, thus reducing motion artifacts [5]. Extension to N beamlines with *N* > 2 is difficult due to spatial and cost constraints [6]. Alternatively, the effective temporal resolution can be improved dramatically either by reducing the effective temporal window of the reconstruction [7, 8] or by estimating the motion and compensating for it in the image generation step [9–11].

Banding artifacts (the second challenge) can be addressed by scanning the entire heart in a single beat. The fastest way to scan a large volume is to use an axial scan with a detector that is sufficiently wide to cover the entire volume at once. Scanners with 16 cm coverage now make this possible for the heart. Another way to cover the entire heart in a single beat (thus avoiding banding artifacts) is to combine a dual-source geometry with a high helical pitch [12]. As the patient is translated through the scanner bore opening at high speed, projection data are acquired through a double helix scan trajectory. The latter approach does have some temporal skew between the base and the apex of the heart and does not allow dynamic (i.e., four-dimensional) imaging.

The fourth challenge (noise) is closely related to X-ray dose reduction since there is a tradeoff between X-ray dose and image noise. Improved temporal resolution and faster gantry speeds have naturally reduced X-ray dose by reducing the scan to a single rotation without the need for additional phase padding. Dose is further reduced by selecting an appropriate kiloVolt peak (kVp) setting, which can now be done automatically. Lower kVp settings (e.g., 80 and 100), which were once uncommon, are now routine for smaller patients thanks to new tube technology. Combining a low kVp and a high milliAmpere (mA) often produces better contrast-to-noise ratio at matched dose than scanning at 120 kVp. In addition, electrocardiogram-based tube current modulation can ensure that the dose is reduced outside the central 180-degree scan range.

The challenge of image noise has also been addressed by the introduction of improved detector readout electronics, new low-signal correction algorithms, as well as non-linear reconstruction algorithms, including fully model-based iterative reconstruction (IR) and, more recently, deep learning (DL)-based reconstruction techniques.

While recent technology introductions have greatly improved banding, motion and noise challenges, progress in improving spatial resolution has been limited and, as a result, the challenge of calcium blooming remains. Stents are also subject to partial volume artifacts, making the identification of in-stent restenosis (ISR) very difficult. While CCTA has a high negative predictive value (NPV) for the identification of obstructive CAD ranging from 84% at worst to 99%, the positive predictive value (PPV) is far lower, ranging from 62% to 87% at best [13–15]. This is largely because many patients have a heavy coronary calcium burden, which can make assessing the severity of a stenosis difficult. In addition, CT is now often used for assessing the functional significance of coronary lesions via computational fluid dynamics. The accuracy of such techniques depends heavily on a careful segmentation of the coronary lumen in the region near potentially flow-limiting stenoses. Such stenoses often contain calcium and the presence of calcium makes segmentation accuracy much more difficult to achieve.

The purpose of this paper is to give a review of the state of the art in addressing the challenge of calcium (and stent) blooming artifacts in CCTA. The paper is organized as follows: Clinical significance of blooming artifacts section gives more detail on the clinical significance of blooming artifacts, root causes of blooming artifacts section discusses the root causes of blooming, solutions for blooming artifacts section gives a survey of methods proposed to address these challenges, and conclusions and future directions section provides some concluding remarks as well as an outlook for future progress in this area.

## Clinical significance of blooming artifacts

### Calcium blooming

**Fig. 1:**
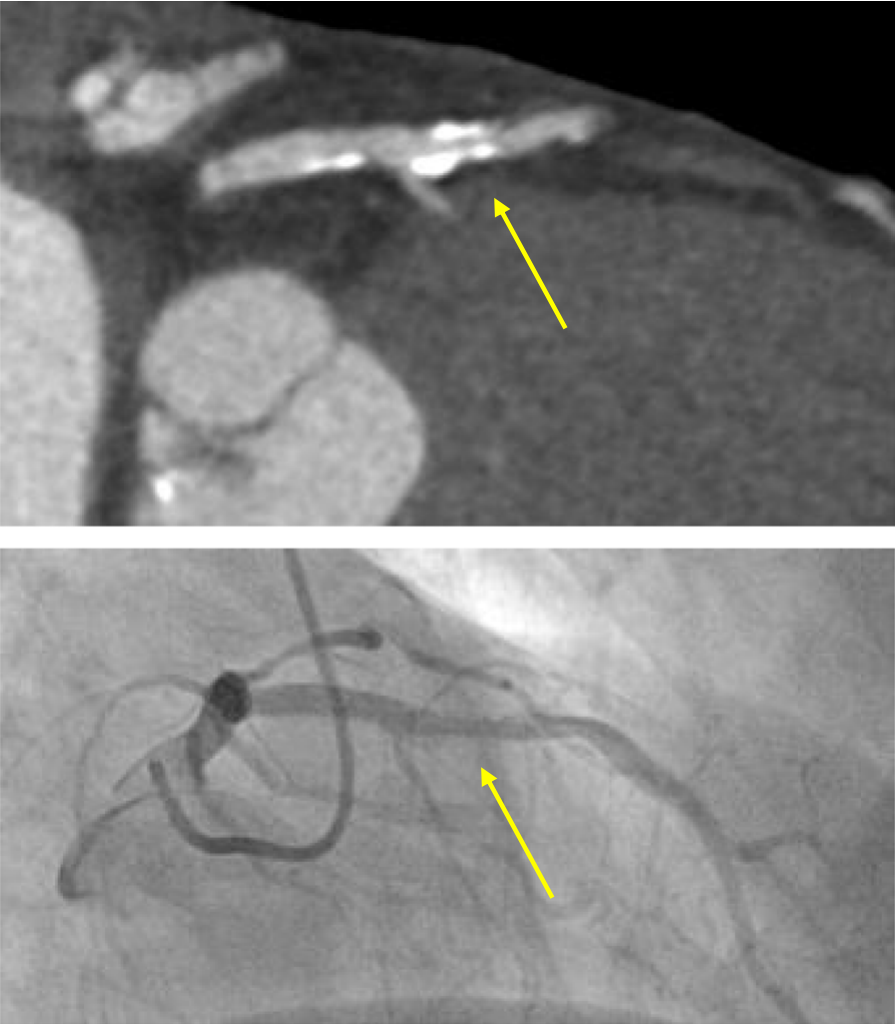
In this example, the mid-left descending coronary artery lesion (yellow arrow) is calcified. Blooming artifact in the CT image results in overestimation of lumen stenosis in this area. Invasive coronary angiography (ICA) revealed a less severe stenosis than perceived on CT. Fractional fluid reserve for this lesion was 0.82. Image credit: Centro Cardiologico Monzino (used with permission)

Clinically, CCTA is used for the identification of significant coronary stenoses in patients and is guideline-recommended as a valuable noninvasive alternative in the diagnostic evaluation of CAD [16, 17]. Despite this, one of the main current limitations of CCTA is inaccuracy in evaluating calcified lesions (Fig. 1).

**Fig. 2:**
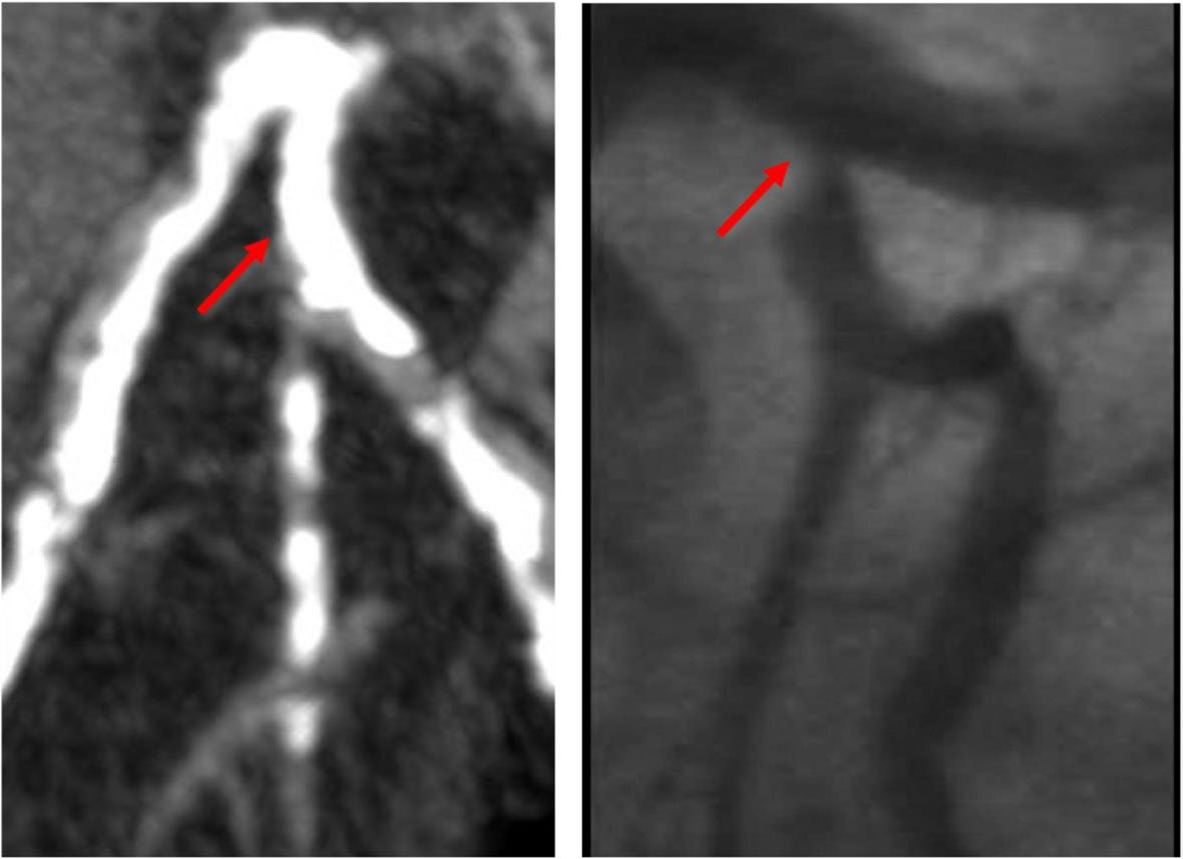
In this example, the proximal part of the left circumflex coronary artery (LCx) has heavy uniform calcification, resulting in blooming in the CT image. This results in the inability to distinguish the severe ostial LCx stenosis (red arrow), that is easily identified using ICA

The presence of calcified lesions leads to blooming artifacts. This may obscure the lumen and cause a false-positive CCTA. Diffuse or extensive calcifications often lead to overestimation or, paradoxically, underestimation of coronary stenosis severity (Fig. 2). Numerous studies comparing CCTA with various invasive methods have shown increased inaccuracy of CCTA with increased calcification.

In a prospective, multicenter study examining the diagnostic accuracy of CCTA in comparison to invasive quantitative coronary angiography (QCA) in 291 patients, using ≥ 50% stenosis as a cutoff, the presence of calcification independently increased the odds for overall misdiagnosis (odds ratio (OR): 2.49; 95%CI: 1.73–3.58), and more significantly, for a false positive (FP) diagnosis (OR: 10.16; 95%CI: 5.23–19.77) at a segmental level. On a patient level, those with a true negative diagnosis had lower coronary artery calcium scores (CACS) compared to those with a FP diagnosis (CACS: 1 vs 49, *p* = 0.047) [18]. In a separate substudy in the same cohort, it was found that the risk of a misdiagnosis is increased by 3.0% for each increase of 10 Agatston units in CACS in a vessel (*p* = 0.002) [19].

In a cross-sectional study comparing CCTA to intravascular ultrasound interrogating 525 coronary lesions, CCTA underestimated lumen area measurement by 5.0%. The relative inaccuracy of CCTA was independently correlated to calcium burden. This was more prominent in vessels with a smaller luminal area, suggesting that artifacts caused even by moderate calcium deposits may turn into critical diagnostic errors. However, no significant relationship was found between high calcium density, a main determinant of blooming artifact, and CCTA inaccuracy [20].

In a meta-analysis of 1634 patients (17943 segments), CACS ≥ 400 was used as a cutoff, and ICA was used as a reference standard. Specificity significantly dropped when comparing high vs low CACS, both on a patient level (OR: 1.40; 95%CI: 1.02–1.93) and segment level (OR: 1.05; 95%CI: 1.00–1.11) (*p* < 0.05 for all). Specificity dropped from 84% to 42%. It is noted that overall sensitivity was not affected [21].

This drop in specificity was concordant with a separate study that showed that calcium affects the overall performance of CCTA, with both a reduction in specificity from 90% (95%CI: 83%–94%) to 44% (95%CI: 14%–79%) as well as a reduction of area under the receiver operator characteristics curve (AUC) from 0.93 (95%CI: 0.90–0.95) to 0.81 for patients with a CACS ≥600 (95%CI: 0.71–0.89, *p* = 0.077 vs < 600) [22].

### Stent blooming

**Fig. 3:**
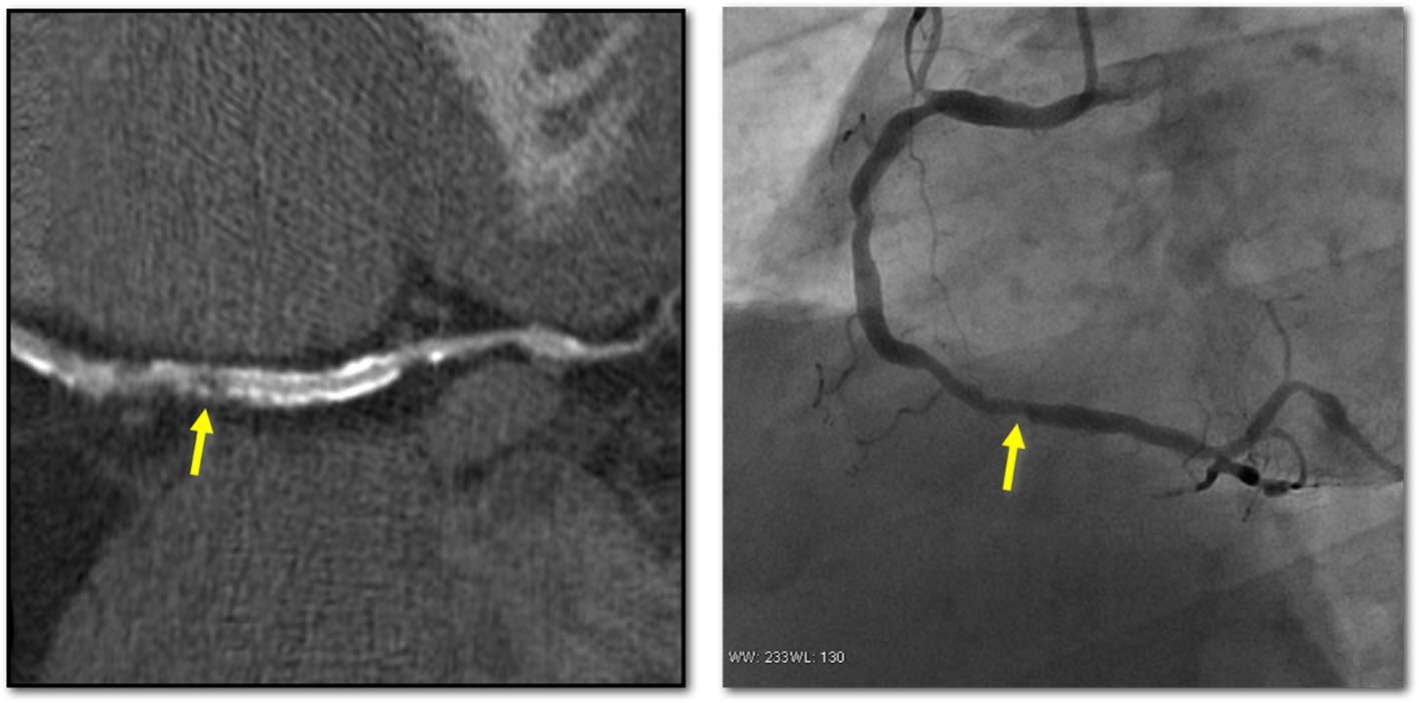
In this CT image, blooming causes overestimation of the severity of ISR in the proximal part of this bare metal stent (yellow arrow). ICA reveals the stenosis to be marginal

The concept of using CCTA for assessment of coronary stents, specifically the occurrence of ISR is inherently attractive. The primary desire is to accurately identify patients who would likely benefit most from percutaneous coronary intervention (PCI) and minimize its use in low-risk patients. However, the same major issues that affect the CCTA assessment of heavily calcified coronary arteries also affect assessment of metallic stents, namely artifacts and blooming of stent struts such that stents appear larger than they actually are, resulting in artificial luminal narrowing and decreased intraluminal attenuation values (Fig. 3).

In a previously mentioned prospective, multicenter study, previous PCI was independently associated with patient-level misdiagnosis (OR: 4.18; 95%CI: 1.56–11.22) [18].

A meta-analysis of 1231 stented (assessable) segments using 64-slice CCTA showed an overall sensitivity of 91% (95%CI: 86–94), specificity of 91% (95%CI: 89–92), PPV was 68% (95%CI: 63–73), and NPV was 98% (95%CI: 97–99) when compared to ICA. However, when including non-assessable segments, performance deteriorated, with a decrease in sensitivity to 87% (95%CI: 81–92), specificity to 84% (95%CI: 82–87), PPV to 53% (95%CI: 47–59), and NPV to 97% (95%CI: 96–98). Increasing stent diameter demonstrated a trend towards more accurate diagnosis (OR: 1.44; 95%CI: 0.87–2.38, *p* = 0.14). Only 5 studies in this meta-analysis included stents with diameters < 3.0 mm [23]. As it has been suggested that 1/3 of unselected patients receive smaller stents of ≤ 2.5 mm, it may be that overall, CCTA may inaccurately assess or be unable to assess a large number of stents [24].

In a study using 320-slice CCTA, for stents with a diameter < 3.0 mm, a significant higher percentage of scans were of poor image quality (35%) as compared with stents with a diameter ≥ 3.0 mm (9%). For stents of diameter ≥ 3.0 mm, the sensitivity, specificity, positive, and NPVs were 91%, 90%, 63%, and 98%, respectively, whereas for stents of diameter < 3.0 mm, the sensitivity, specificity, positive, and NPVs were 100%, 63%, 13%, and 100%, respectively. Diagnostic accuracy also deteriorated with decreasing stent size, from 90% to 65% (*p* = 0.019). Stent strut thickness also affected the diagnostic performance of CCTA. The sensitivity, specificity, positive, and NPVs of CCTA for strut thickness < 140 μm were 89%, 86%, 47%, and 98% respectively. However, this deteriorated to 100%, 50%, 20%, and 100% respectively for stents with a strut thickness ≥ 140 μm [25]. This pattern of CCTA performance deterioration with decreasing stent diameter and increasing strut thickness was also echoed in another meta-analysis. Additionally, this study found higher sensitivity and specificity in simple stenting than in bifurcation stenting (*p* = 0.012 and *p* = 0.001, respectively) [26].

Modern IR methods have been partly successful in reducing this drop in performance. In a study comparing IR to filtered backprojection (FBP), IR trended towards improvement (85% vs 85% sensitivity, 89% vs 78% specificity, 73% vs 57% PPV, 95% vs 94% NPV, and 0.87 vs 0.82 AUC), although these improvements did not reach statistical significance. However, in stents of ≤ 3 mm diameter, IR performed better than FBP with specificity (82% vs 62%), PPV (66% vs 50%) and AUC (0.81 vs 0.70) all significantly improved [27]. Subtraction CCTA also improved performance compared to conventional CCTA, reducing the FP rate in stented segments from 85% (95%CI: 71–95) to 19% (95%CI: 9–34) [28].

As blooming artifacts exaggerate the size of dense calcium, they impede the accurate evaluation of the adjacent lumen, with a resultant overestimation of lesion severity. A consequent increase in FP rate may also decrease specificity, often leading to unnecessary ICA to exclude stenotic CAD, which incurs additional cost, patient radiation exposure, and potential complications, thus limiting the clinical usefulness of CCTA [29].

In coronary stent evaluation, beam hardening and blooming may have downstream clinical workflow and decision-making consequences. As a result of these artifacts obscuring part of the stent lumen, artificial lumen narrowing is observed. In the case of a smaller stents, larger proportion of the stent lumen may be obscured, thereby rendering the image uninterpretable. Optimal CCTA stent image quality is only achieved in patients with large stent diameter and thin stent struts. Accordingly, careful patient selection regarding stent type, diameter, and strut thickness is required. Regardless, the use of CCTA as a gatekeeper for ICA, in symptomatic patients with a history of stent implantation is extremely limited as a consequence.

## Root causes of blooming artifacts

Blooming of calcium and stents has primarily been attributed in the scientific literature to three different root causes: partial volume averaging, motion, and beam hardening [30–32]. From our survey of the literature, we have compiled a table showing the frequency with which each effect is mentioned in connection with calcium blooming (Table 1). The most commonly cited factor is partial volume averaging due to the limited spatial resolution of the scanner. Partial volume artifacts make it difficult to cleanly delineate the edges of high contrast objects. Uncorrected motion artifacts can also obscure the boundaries of dense objects and result in a loss of confidence in segmenting the vessel lumen. Consequently, several authors categorize motion as a source of blooming, while others refer to motion blurring as a separate type of artifact. Regardless, motion certainly obscures the calcium boundaries and thus contributes to degrading the PPV of CCTA for patients with a high calcium burden. The third factor (beam hardening) is a bit more controversial, having been blamed by some for blooming, while being cleared by others. We will discuss each of these three artifact mechanisms separately in a subsection below along with some of our own findings with respect to beam hardening and motion.

**Table 1:** Overview of literature citing various potential root causes of calcium blooming

Root causes	Related references
Partial volume	[30–41]
Motion	[28, 31–33, 37]
Beam hardening	[31, 32, 34, 38–41]

The most cited factor is partial volume averaging, followed by motion artifacts and beam hardening artifacts

### Partial volume averaging

Partial volume averaging is the primary cause of calcium blooming. There are five different factors that contribute to this blurring: detector cell size, focal spot size, azimuthal blur, crosstalk, and the reconstruction algorithm/kernel [42]. Modern CT scanners typically have physical detector cell sizes of approximately 1 mm in both height and width, which corresponds to a size of roughly 0.6 mm at the scanner isocenter (after accounting for the system magnification). The X-ray focal spot size varies, but for a high mA scan it is similar to the detector cell size after projecting both to the isocenter. Another source of blurring that can be quite significant when the heart is not well centered in the scanner is the azimuthal blur, which stems from the gantry rotation that occurs during a single view. Detector crosstalk (including X-ray cross-talk, optical cross-talk and electronic cross-talk) can also contribute, although these effects are usually small. The final source of blurring is the reconstruction algorithm itself, which can include interpolation during fan-to-parallel rebinning and backprojection as well as intentional low-pass filtering designed to reduce noise and aliasing. It should be noted that the filtering kernel can also be boosted at mid-range spatial frequencies to partially compensate for the blurring induced by some of the physical blurring mentioned above (at the expense of higher noise and aliasing).

It is important to consider how much spatial frequency information is preserved in the sinogram data itself, as no changes to the reconstruction algorithm can hope to recover missing data without some amount of a priori assumptions. The cutoff frequency near the center of the field of view is constrained by the blurring from the finite detector cell size. One can model the effect of the blurring that the finite detector cell size induces in the projection data as a convolution of an ideal projection with the sensitivity function of a single detector pixel, followed by a sampling with a sampling interval equal to the cell spacing. If the detector sensitivity function is modelled as a rect function of width 0.6 mm, a periodic fluctuation in the ideal incident X-ray intensity with a period of 0.6 mm would be eliminated after convolution with the detector sensitivity function (without even considering the other sources of blurring mentioned above). This corresponds to a cutoff frequency of approximately 17 lp/cm (line-pairs per centimeter). In practice, the active area of the detector is a bit smaller than this (at isocenter), which allows frequencies that are a bit higher than 17 lp/cm to be reconstructed with the sharpest of kernels. We note that focal spot deflection (or wobble) can improve the sampling frequency, but that it does not increase the cutoff frequency (it does reduce aliasing and enables sharper kernels). Importantly, no data processing technique can recover frequencies (well above 17 lp/cm) that have already been eliminated from the projection data due to the finite system resolution, at least not without some prior knowledge or assumptions about the object being imaged [43].

**Fig. 4:**
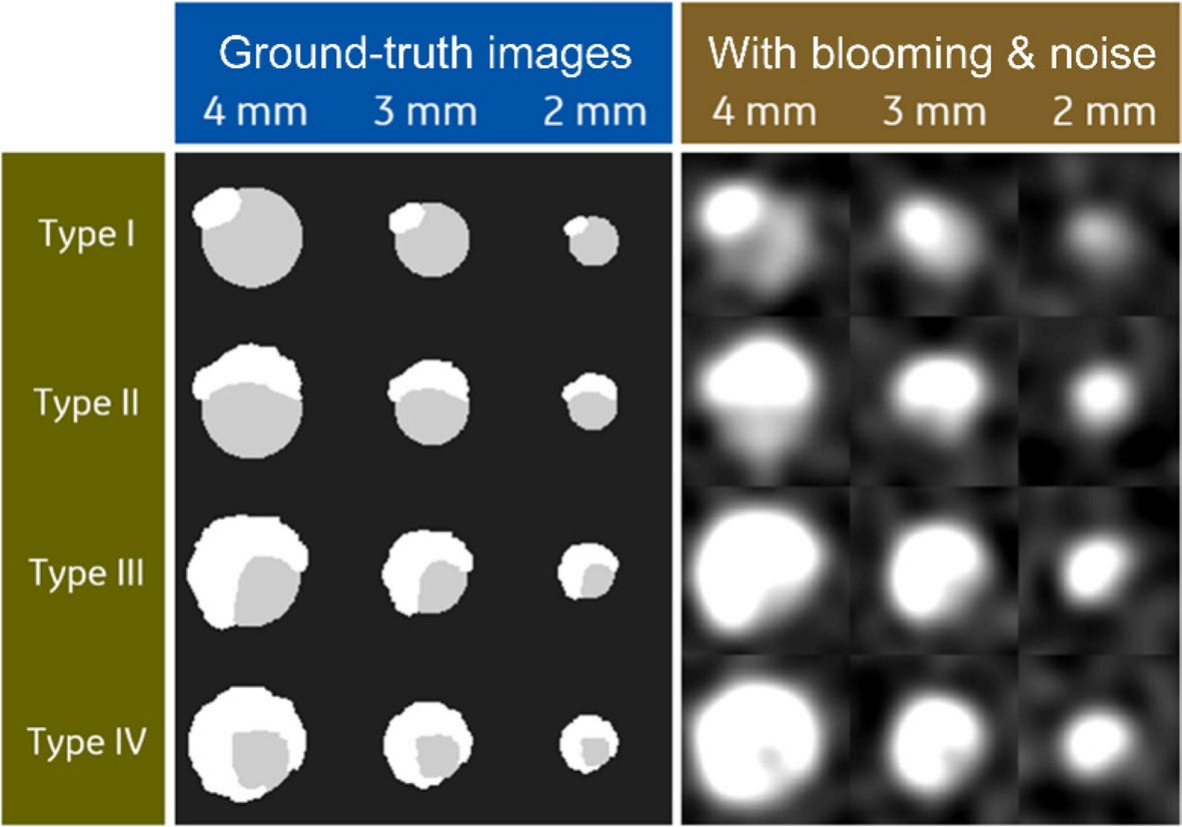
Four plaque types (as defined by Qi et al. [13]) are simulated here in 3 sizes (with 4, 3, and 2 mm unobstructed diameters). The calcium (white, 1750 Hounsfield units (HU)) is blurred by the system point spread function such that it obscures much of the lumen (light gray, 600 HU) in many cases. The original (ground truth, high resolution) images are shown in the left panel. The right panel simulates the effects of finite system resolution and noise (W/L = 1000/300 HU)

Coronary calcifications vary greatly in size but the most clinically relevant ones for assessing vessel patency are approximately 1–3 mm in diameter. Blurring with the system point spread function described above causes these calcifications to appear larger than they really are. This effect is more pronounced for denser calcifications and also when viewed with a narrow window/level setting that is tuned for looking at the contrast enhanced lumen and the surrounding soft tissue. Figure 4 illustrates this problem.

### Motion artifacts

**Fig. 5:**
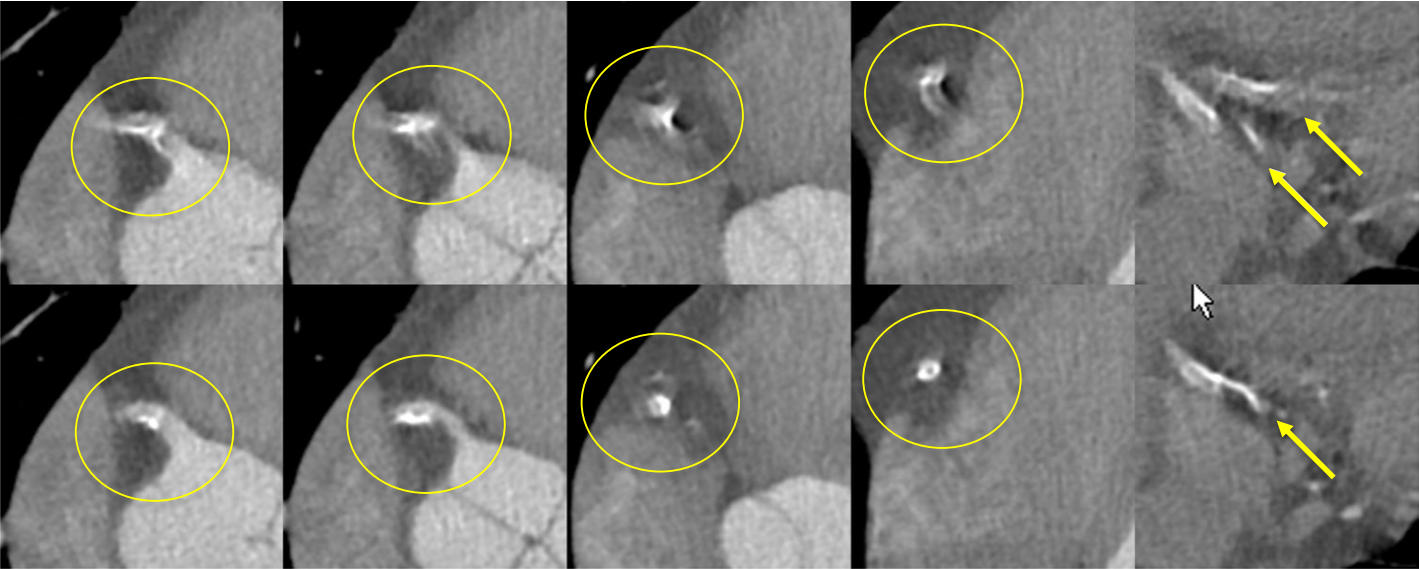
Axial slices from the RCA of a patient with heart rate of 86 bpm illustrate strong motion artifacts (top). After algorithmic motion correction using SnapShot Freeze 2 (bottom) the artifacts are reduced substantially, leading to more interpretable images. The images are ordered from proximal to distal. W/L = 1100/100 HU (Credit: Anzhen Hospital)

Motion artifacts, if not corrected, can be a serious issue in CCTA. These artifacts result from data inconsistencies, which violate the assumptions of traditional reconstruction approaches and are produced by involuntary motion of the beating heart [42]. Motion artifacts are particularly problematic for small, fast moving structures, so the apparent shape and clarity of coronary calcifications can often be substantially degraded. Fortunately, as mentioned previously, motion artifacts can be corrected algorithmically. Figure 5 shows an example of algorithmic motion artifact correction (SnapShot Freeze 2 is used for motion correction throughout this paper) near calcified plaques in the right coronary artery (RCA). After correction, the shape of calcium and the lumen boundaries are much clearer. Vessels that are oriented such that their centerline is nearly parallel to the scan plane are susceptible to severe motion artifacts if the gantry angle happens to be such that rays align with the centerline twice during the half-scan time window: once near the beginning and once near the end. Without correction, such vessels (or sections thereof) can essentially be duplicated in the image and appear broken (e.g., top-right panel of Fig. 5). With advanced motion correction algorithms, the vessel shape is well restored (e.g., bottom-right panel of Fig. 5).

### Beam hardening

As X-rays from a polychromatic beam pass through the body, those with low energy are preferentially attenuated making the overall beam ‘harden’ such that it contains a larger portion of high energy photons when compared to the incident beam [33]. This happens because the attenuation coefficient of X-rays with high energy is smaller than that of X-rays with low energy (for most clinically relevant energies and materials). In the absence of beam hardening (e.g., if we had a monochromatic energy for the X-ray beam), there would be a linear relationship between the attenuation path length and the log of the ratio between the incident and transmitted X-ray intensity. The non-linearities introduced by beam hardening are easy to correct if the object being scanned is predominantly composed of water or other soft tissues with a similar effective atomic number, but there can be residual errors if the object contains a lot of a material that has an effective atomic number that is very different than that of water. Modern reconstruction algorithms can typically mitigate the effects of beam hardening even for the multi-material case (in this paper we used the multi-material beam-hardening correction from the GE Revolution CT scanner).

There are several compelling arguments that can be used to implicate beam hardening as a source for calcium and stent blooming. Firstly, calcium and stents contain materials with high effective atomic numbers, including calcium and phosphorus in the case of calcified plaque and iron and/or cobalt (or even tantalum) in the case of stents [33]. Metal artifacts from surgical wires or clips, dental fillings, and artificial joints are known to be due in part to beam hardening. Also, it has been noted that using a higher kVp leads to less apparent blooming when images are compared on a fixed HU window [33]. Finally, the characteristic appearance of some of the artifacts that seem to spray off of calcifications and stents is similar to what is observed in beam hardening induced errors elsewhere in the body coming from dense bones or metal objects: alternating bright and dark streaks coming off preferentially in certain directions [42]. Such streaks can cause the contrast enhanced lumen to have areas that appear too dark and this can sometimes mimic the appearance of soft plaque.

Skeptics of the influence of beam hardening on calcification and stent blooming would point out that, while coronary calcifications and stents do have a high effective atomic number, they are very small (calcifications are no more than a few millimeters and stent struts, though dense, are measured in microns) in comparison to the objects that typically cause residual beam hardening effects (e.g., contrast enhanced blood pools and large bones in the shoulders, hips, or head) and could not cause a significant shift in the effective energy of the X-ray beam. (The orientation of the stent should also be considered since, in rare cases, a steel or tantalum-core stent can be nearly parallel to the scan plane such that a small set of X-ray paths will pass through many, many stent struts.) Furthermore, the reduction of blooming at higher kVp (or at high monochromatic kilo-electron-Volt image setting with dual energy) can easily be attributed to the fact that the HU scale only compensates for the attenuation difference of water and not denser materials like iron and calcium. One could make a case that using a higher energy beam is not unlike simply using a wider window setting to review the images. In either case, there appears to be less blooming, but the contrast between the lumen and the background will be reduced. In fact, a common strategy for mitigating the effects of calcium blooming is to use a wider window for assessing regions that are impacted by blooming [33]. With respect to the characteristic appearance of bright and dark streaking, it should be appreciated that motion artifacts can often have a similar appearance. It is quite easy for residual motion artifacts around dense structures to be incorrectly attributed to beam hardening [33].

**Fig. 6:**
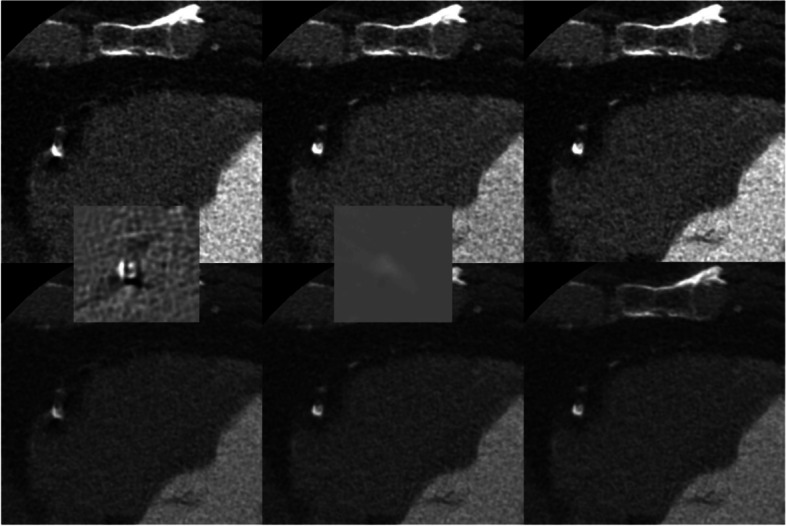
CCTA exam (70 bpm, 70% R-R phase) with bright and dark streaks from calcification and effect of motion correction and beam hardening correction. Left: No motion correction, with multi-material beam hardening correction; Center: With motion correction, no multi-material beam hardening correction; Right: Fully corrected (for both motion and beam-hardening). Left inset: Difference image (with vs without motion correction); Center inset: Difference image (with vs without multi-material beam hardening correction). Top row W/L: 900/200; Bottom row W/L: 1600/400; Insets (W/L = 900/0) (Credit: UCSD)

To test the impact of beam hardening and compare it to the impact of motion, we found an image with bright and dark streaks emanating from and obscuring the boundary of a coronary calcification. We compared fully corrected images to those without beam hardening and also to those without motion correction. Figure 6 illustrates the results. It is clear that, at least in this case, motion correction has a much bigger impact than the multi-material beam hardening correction near the calcification.

**Fig. 7:**
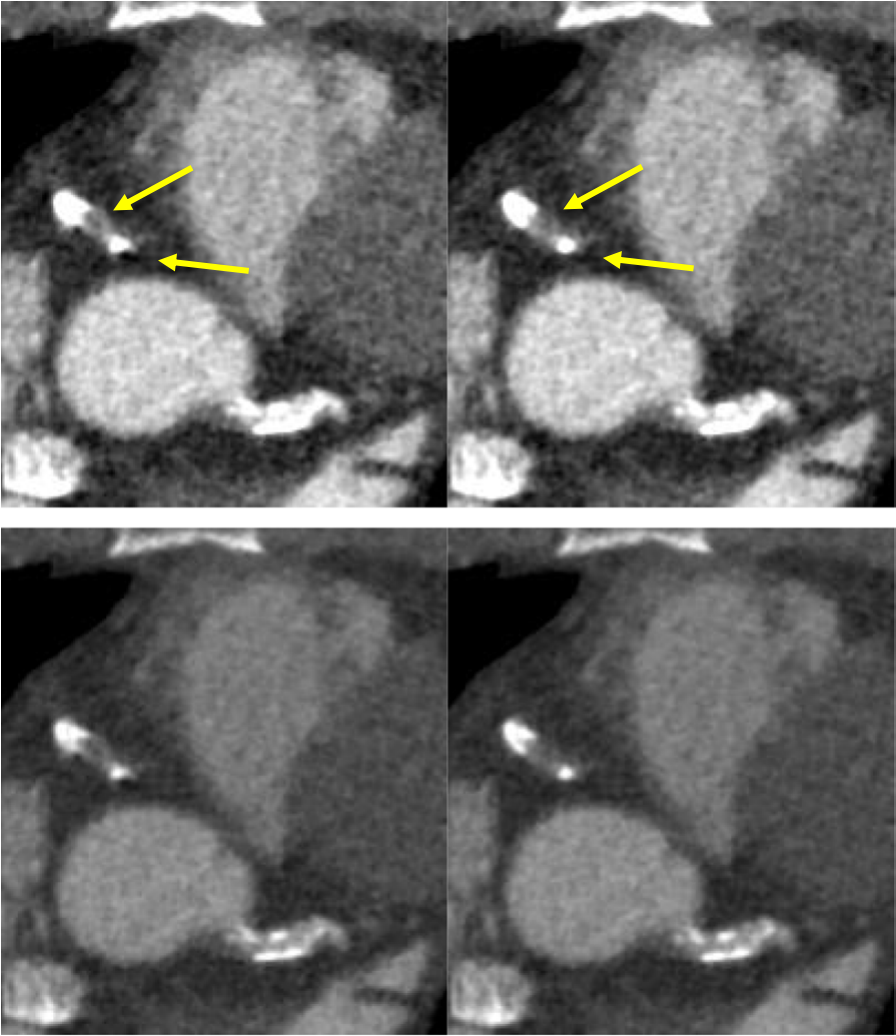
CCTA exam (63 bpm, 70% R-R phase) with two calcifications and the effect of motion correction. Left: Without motion correction; Right: With motion correction. Top row W/L: 900/200; Bottom row W/L: 1600/400 (Credit: UCSD)

We also found a case in which a dark streak between two calcifications in the uncorrected images gave the appearance of possible soft plaque. After applying motion correction, this artifact is mitigated (Fig. 7). These are clear indications that the beam hardening effect of calcifications is very small and essentially absent, unlike the motion artifacts, which are easily confounded with beam hardening artifacts. Both cases (Figs. 6 and 7) also illustrate the importance of choosing an appropriate window width for viewing calcifications in order to mitigate the blooming effect.

In summary, partial volume averaging is the primary root cause of blooming. Motion artifacts can also cause calcium and stent struts to bloom beyond their true size, but in scanners with advanced motion correction, this effect is usually well corrected. Beam hardening has, in the past, been considered as a root cause for blooming, but with modern CT scanners, the impact of even a relatively large piece of coronary calcium or a coronary stent strut on beam hardening is minimal.

## Solutions for blooming artifacts

**Fig. 8:**
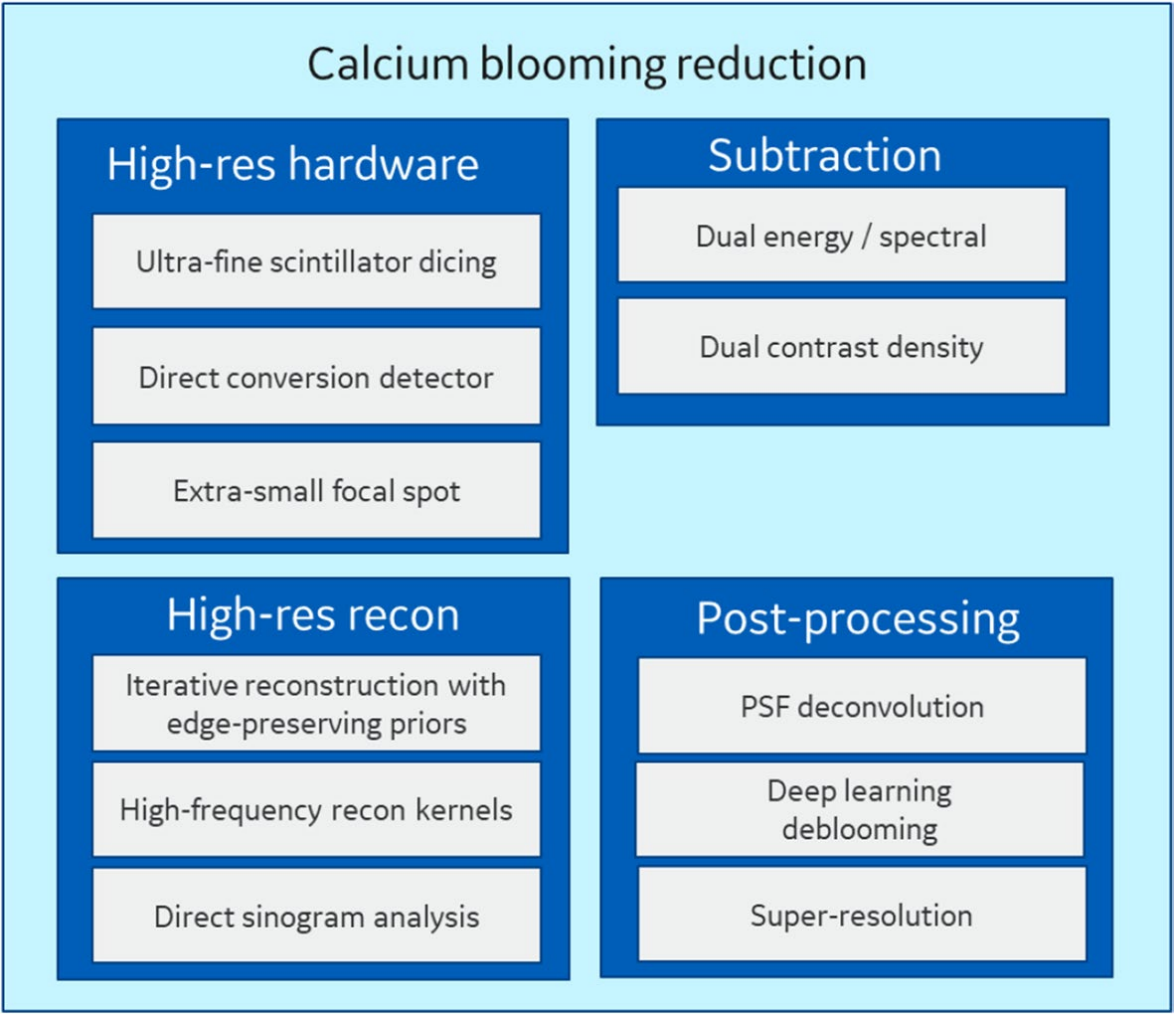
A diagram illustrating various technical approaches for mitigating the primary cause of calcium blooming: partial volume averaging

In this section we detail four broad approaches for reducing blooming of calcium and stents from the published literature. For the most part, these approaches are targeted at addressing partial volume averaging, but one should keep in mind that motion artifacts must also be addressed. Some of these techniques can exaggerate motion artifacts, but even without this, the solution to any one artifact in CT tends to make all other artifacts more apparent. An overview of calcium blooming reduction approaches found in literature is shown in Fig. 8. The first broad approach for deblooming is the direct approach: modifying the scanner to enable higher resolution. Second, there are new reconstruction techniques which use non-linear techniques to boost resolution near dense structures, while suppressing background noise at other locations. Third, there are subtraction techniques, wherein the dense structure is removed either through a material decomposition technique from a dual energy scan or by leveraging two scans with different amounts of iodine contrast. Finally, there are image based post-processing techniques, some of which leverage DL with convolutional neural networks to deblur the calcium or to replace it with a lower density tissue intensity.

### High resolution CT hardware

Given that the primary cause of blooming is the finite impulse response of the CT system, producing a CT scanner with a denser detector pixel grid and a smaller focal spot is a good way to reduce blooming [34]. While such scanners are available, they are currently rare. In addition, many researchers and CT vendors are currently exploring or developing new photon counting detector technologies, some of which have significantly higher spatial resolution capabilities than today’s clinical scanners [44, 45]. Photon counting (direct conversion-based) detectors have an inherent detection efficiency advantage over light scintillator-based detectors that becomes more pronounced at higher resolutions because the effective active area of a scintillator cell is reduced due to dicing, optical reflectors, and X-ray shielding. On the other hand, photon-counting detector technology is still suffering from several challenges, including sensor impurities and instabilities, charge sharing, and pulse pile-up at high count rates, which limit its applicability for cardiac CT due to the high scanning speeds involved. So, it is currently unclear how quickly ultra-high-resolution technology will become mainstream for cardiac scanning. Ultimately, the benefits of higher resolution must be weighed against cost. In addition to the initial cost of a CT scanner, for which detector cell count is a major driver, a double resolution scan can produce image volumes that require 8 x more memory to cover the same volume, which requires more resources to store, transfer and diagnose. Also, CCTA is an application that requires more than just spatial resolution. Though it would be costly, an ideal cardiac scanner would combine ultra-high spatial resolution with other critical cardiac imaging capabilities, including large longitudinal coverage, fast gantry rotation, and accurate motion correction.

### High resolution reconstruction

A second type of approach is to modify the reconstruction to preserve resolution as much as possible. When a non-linear reconstruction is used, image priors can be incorporated to preserve or even enhance sharp edges. At the same time, background noise away from the edges can be suppressed [13]. In one study only including patients with CACS ≥400 and ICA as a reference, IR improved specificity and PPV over FBP, both on a patient and segment level [36]. Optionally, two reconstructions can be performed: a standard one and a high resolution/denoised one. Blending can then be performed to use the standard image in the bulk of the volume while blending in the high-res image only near the dense structures (e.g., calcium and stents) [37]. This type of approach is quite valuable since the use of a softer reconstruction kernel is primarily driven by the desire to limit noise in the low contrast/background regions. At the same time, there is a limit to this approach since the inherent system resolution is unchanged. Finally, reconstruction can be bypassed entirely by analyzing projection data directly, avoiding any reconstruction-induced blurring effects [46].

### Subtraction techniques

Subtraction techniques are based on the idea that if we can find a way to remove dense structures from our image entirely, spatial resolution becomes much less important to the assessment of luminal diameter and patency. The key is to find some way in which the lumen and the dense structure can be separated in the data.

One way to separate dense structures from the lumen is to use dual energy data and then to use material decomposition either to transform to a material basis image in which the dense material is suppressed or to quantify the volume fraction of the dense object in each voxel and subtract it out. One risk with this approach is noise amplification through the material decomposition process. Also, if there is any significant time skew between the acquisition of the two energy bins, there can be misregistration errors that will be amplified by material decomposition. Only scanners with dual-layer detectors, photon-counting detectors and fast kVp switching avoid this time skew and effectively enable subtraction.

A second way to separate the dense structures from the lumen is through two scans with different levels of contrast. By subtracting the two scans, the hope is to isolate the lumen, thus producing a calcium/stent free view to facilitate diagnosis. As noted by Razeto, however, “precise registration of structures is essential, as even small misalignments can produce striking and disruptive bright and dark artefacts” [47]. Furthermore, any motion artifacts in either of the two initial images will manifest themselves in the subtracted image. Still, this technique has shown good results for carefully selected segments. In one study, subtraction improved the AUC from 0.74 (95%CI: 0.60–0.89) to 0.91 (95%CI: 0.79–1.00, *p* = 0.003) when compared to QCA [48]. In a later, prospective, international, multicenter study using ICA as reference, coronary subtraction software reduced the FP rate from 65% to 41%. However, there was a high rate of misregistration, resulting in exclusion from software application [28].

### Image-based post-processing techniques

Several image-based post-processing techniques have been proposed. For example, deconvolution using a measured point spread function has been shown to reduce blooming [31, 49]. Other post-processing techniques leverage DL, which is a branch of machine learning that has shown incredible capability in image processing. The Unet network architecture can be used to fuse features identified at several different scales and are effective for image processing applications like this [50]. DL networks can be trained either to sharpen calcium or to remove it altogether [38] depending on the type of images used to train them. One important consideration for post-processing algorithms is to maximize the amount of information that is preserved through the reconstruction process [51]. This may require the initial reconstruction to have a dense pixel sampling. High frequency noise and aliasing can be removed in postprocessing. On the other hand, if there are spatial frequencies that are eliminated in the recon, they may not be faithfully recovered. Generally, since we established above that partial volume blur is the dominant cause of blooming artifacts, approaches that target super-resolution or deblurring may also prove effective to reduce blooming artifacts. In recent years, numerous DL networks have been shown to greatly enhance image sharpness and reduce spatial blur [52–55]. While these approaches were originally designed for more general deblurring purposes, they could be retrained and selectively applied in patients and locations with calcium deblooming artifacts or stent deblooming artifacts.

## Conclusions and future directions

While CCTA imaging has been greatly improved in terms of volume coverage and motion avoidance/correction, progress in improving spatial resolution has been limited and, as a result, the challenges of calcium blooming and stent blooming remain. As blooming artifacts exaggerate the size of dense calcium and stent struts, they impede the accurate evaluation of the lumen, with a resultant overestimation of lesion severity and ISR.

Blooming artifacts have been attributed to the partial volume effect, motion artifacts as well as beam hardening. Partial volume averaging is the primary root cause of blooming. In the absence of advanced motion correction, residual motion artifacts can also cause calcium or stents to bloom beyond their true size. With modern CT scanners, the role of beam hardening in calcium blooming is minimal.

Solutions to blooming can be categorized as high-resolution CT hardware and reconstruction, two-pass subtraction techniques and post-processing techniques. We conclude that blooming can partially be prevented by increasing the CT spatial resolution through higher-resolution CT hardware or advanced high-resolution CT reconstruction. We expect future progress along this direction to be somewhat limited as long as the physical size of detector pixels in common clinical practice remains at approximately 1 mm. We expect future work in subtraction techniques based on two scans to be limited due to the inherent challenges, but those based on temporally registered dual energy will benefit from recent and future progress in noise reduction techniques. DL techniques have shown great promise to correct for blooming artifacts and we see this as an important direction for future study. A combination of these techniques could effectively suppress calcium blooming while avoiding repeat scans.

## Data Availability

All data generated or analyzed during this study are included in this published article.

## Abbreviations

CCTA: Cardiac CT angiography
CAD: Coronary artery disease
QCA: Quantitative coronary angiography
CACS: Coronary artery calcium scoring
FP: False positive
ICA: Invasive coronary angiography
RCA: Right coronary artery
AUC: Area under the curve
PCI: Percutaneous coronary intervention
KVp: KiloVolt peak
mA: milliAmpere
PPV: Positive predictive value
NPV: Negative predictive value
FBP: Filtered backprojection
HU: Hounsfield units
DL: Deep learning
LCx: Left circumflex coronary artery
OR: Odds ratio
CACS: Coronary artery calcium scores
IR: Iterative reconstruction
ISR: In-stent restenosis
